# Optical Fiber Delivered Ultrafast Plasmonic Optical Switch

**DOI:** 10.1002/advs.202100280

**Published:** 2021-03-16

**Authors:** Jinghui Yang, Xinping Zhang

**Affiliations:** ^1^ Institute of Information Photonics Technology Faculty of Science Beijing University of Technology Beijing 100124 China; ^2^ Modern Police Technology and Equipment Research Center College of Police Equipment and Technology China People's Police University Langfang 065000 China

**Keywords:** end facets, flexible transfer, higher‐order localized surface plasmons, integration with optical fibers, plasmonic nanostructures, ultrafast optical switch

## Abstract

Ultrafast optical switch based on plasmonic nanostructures has large application potentials in optical logic circuits and optical communication systems. Integration of plasmonic optical switching devices with optical fibers is a breakthrough for realizing practical applications in long‐range optical data transmission or communication techniques. Here, the incorporation of plasmonic optical switch devices onto the end facets of optical fibers is reported, so that the switched optical signals are generated by interaction between femtosecond laser pulses and plasmonic nanostructures on one end of the fiber, and are delivered via the fiber waveguide to the other end for detection or decoding. “Quenching” of localized surface plasmon in the gold nanowires by its interaction with band‐edge modulation in gold through strong optical excitation is the responsible photophysics. This work accomplishes for the first time the implementation of ultrafast plasmonic optical switch device on optical fiber tips for logic data transmission.

## Graphical abstract

Plasmonic optical switch consisting of gold nanowire (AuNW) grating is integrated onto the end facet of an optical fiber. The switched optical signals are generated by interaction between femtosecond laser pulses and AuNWs, which are delivered by the fiber waveguide to the unstructured end for detection/decoding. Band‐edge modulation in gold through strong intraband optical excitation is the responsible physics.

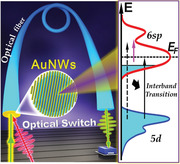

## 1 Introduction

Interaction between ultrashort laser pulses and metallic nanostructures induces multifold photophysical processes, which may involve inter‐ and intraband electronic transitions, band‐edge modulation of the plasmonic electron oscillation, and spectroscopic redshift of localized surface plasmon resonance (LSPR). All of these interactions can be utilized to achieve ultrafast optical switching. High‐efficiency and low‐threshold optical modulation devices are urgently demanded by photonic integrated circuits and optical communication systems, in particular for the applications of on‐chip integration techniques. Various materials, including organic and inorganic dielectrics or semiconductors, e.g., metal–organic frameworks^[^ ^1^ ^]^ and graphdiyne,^[^ ^2^ ^]^ also exhibit excellent nonlinear optical response and can be utilized in high‐performance optical modulation devices. However, the plasmonic devices using metallic micro/nanostructures involve different photophysics and different advantages in applications. High thermal and chemical stability, ultrafast photoelectronic response, and long working lifetime are unsubstitutable properties of plasmonic metallic devices. All‐optical switches based on metallic nanostructures constantly arose with the ultrafast response time scale of surface plasmon resonance.^[^ ^3^, ^4^, ^5^, ^6^, ^7^, ^8^ ^]^ Surface plasmon polaritons (SPPs)^[^ ^9^, ^10^ ^]^ and localized surface plasmons (LSPs)^[^ ^11^, ^12^ ^]^ have been the main mechanisms. The extremely short lifetime, ultraintensive local field modulation within extremely small volumes, and strong nonlinear optical response based on plasmonic electron oscillation are the special advantages of the ultrafast plasmonic optical switching (POS) devices.^[^ ^13^, ^14^, ^15^, ^16^ ^]^ Electron–electron, electron–phonon, and phonon–phonon interactions are generally observed in the optical switching signals. However, only the electron‐involved plasmonic processes can be utilized to achieve optical switching processes with a response time scale ranging from 100 fs to a few ps.^[^ ^17^, ^18^, ^19^ ^]^ Generally, high on/off speed, large spectral shift and consequently large modulation depth, high signal contrast, and broad‐band acceptability or tunability are always expected for the ultrafast optical switching devices. Designs of novel nanostructures and devices have been reported with various periodic and nonperiodic configurations.^[^ ^20^, ^21^, ^22^, ^23^, ^24^ ^]^

Data transmission and communication with controlled logics are the principal applications of all optical switches. Therefore, integration of nonlinear optical materials or optical switch devices with optical fibers^[^ ^25^, ^26^ ^]^ is of more practical importance, which may be directly involved in optical remote sensing, data transmission, and optical communication systems. Although optical switching devices on planar substrates have been reported extensively, fiber‐end‐based plasmonic optical switching techniques or devices have not yet been reported so far. We demonstrate here an all‐optical switch integrated on the end facet of an optical fiber, where the optical switching device consists of periodically distributed gold nanowires (AuNWs) with a thickness of about 200 nm and a width of about 180 nm. Such an integration does not only imply compact optical logic circuit systems, but also mean potential all‐fiber‐based optical modulation for optical communication systems, where fiber lasers can be employed as the driving source.

Fabricating metallic nanostructures on optical fiber tips to miniaturize device and to integrate multifold optical functions has been a most important aspect of “lab‐on‐fiber” techniques.^[^ ^27^, ^28^ ^]^ Although various fabrication methods have been proposed using electron beam lithography,^[^ ^29^, ^30^ ^]^ focusing ion beam lithography,^[^ ^31^, ^32^ ^]^ interference lithography,^[^ ^33^ ^]^ and nanoimprinting,^[^ ^34^, ^35^ ^]^ flexible transfer^[^ ^36^, ^37^ ^]^ is a more effective technique in its high reproducibility, high quality sustainability, and high success rate. In this work, we achieved the fiber‐end‐based optical switching device using the flexible transfer technique, which is based on the quadrupolar localized surface plasmons in the thick gold nanowires. This lays a most important basis for optically switched plasmonic fibers for optical communication applications.

## 2 Design and Fabrication of Plasmonic Optical Switch on the Fiber End Facet

### 2.1 Design and Basic Principles

**Figure 1:**
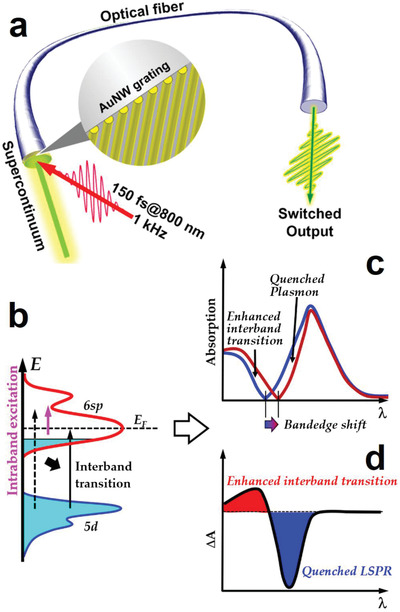
a) Schematic illustration of the fiber‐end‐integrated plasmonic optical switching device, which is characterized by femtosecond pump–probe spectroscopy. b–d) Basic principles for plasmonic quenching as the main mechanism for the optical switching effect.

**Figure** **1** shows schematically the design and basic principles of the optical switching device integrated onto the end facet of an optical fiber. An AuNW grating was transferred from a planar substrate to the end facet of an optical fiber using the flexible method that we reported in our previous publications^[^ ^36^, ^37^ ^]^ and is described in detail in Section 2.2. This process implemented the integration of plasmonic nanostructures onto the fiber tips and finished the realization of fiber‐delivered POS device.

The design of the optical fiber delivered POS is shown in Figure 1a, where femtosecond laser pulses at 800 nm with a pulse length of 150 fs and a repetition rate of 1 kHz were sent onto the AuNW grating with an angle of about 15° with respect to the axis of the optical fiber and were employed as the pump for the optical switching process. Part of the 800 nm pulses was focused into a cuvette containing heavy water with a thickness of about 3 mm to produce supercontinuum pulses, which were focused into the fiber roughly along the fiber axis for probing the optical switching signals. The supercontinuum spectrum extends from about 340 to 1000 nm. The pump beam has a diameter larger than that of the optical fiber, in contrast, the probe beam (<200 µm in diameter) is smaller than the fiber core (400 µm in diameter), so that homogeneous interaction between the pump and probe pulses can be achieved on the AuNWs. The switched probe pulses propagate along the optical fiber and are coupled out of the fiber at the other end after long‐distance travelling, which carry the optical switching signals.

The photophysical principles for the optical fiber delivered POS are interpreted in Figure 1b. Strong optical excitation by femtosecond laser pulses induces strong intraband transition (upward arrow in magenta), leaving a transient depletion band underneath the Fermi level, as shown in Figure 1b. Thus, the originally forbidden interband transition (dashed upward black arrow) becomes allowed to the depleted band (solid upward black arrow). This is equivalent to the lowering of the threshold for interband transitions, as indicated by the downward black arrow in Figure 1b, resulting in the redshift of the band‐edge, as shown in Figure 1c. Such band‐edge shift not only induces enhancement in the interband transitions, but also “destroys” the plasmonic electron oscillation on the lower edge of the plasmonic band, leading to a “quenching” effect, as depicted in Figure 1c. This quenching behavior results in a “left‐positive” and “right‐negative” transient absorption (TA) spectrum, as illustrated schematically in Figure 1d. The left‐positive spectrum is attributed to the redshift of the falling right‐edge of the interband absorption spectrum, due to the optical excitation induced redshift of the threshold for interband transition. Meanwhile, this redshift pushes the rising left‐edge of the plasmon band to the red, leading to the quenching of the plasmonic optical extinction within the corresponding spectrum. This explains the right‐negative TA signal. Therefore, optical absorption is transiently enhanced in the spectral band highlighted by the red‐filled region and optical transmission is enhanced in that designated by the blue‐filling, as shown in Figure 1d. Positive and negative optical switching behaviors can be achieved in the red‐ and blue‐filled spectral regions, respectively. These mechanisms supply new photophysics for optical switching and are verified convincingly by our experimental results in the following sections. Moreover, such an ultrafast POS device is integrated with an optical fiber, enabling much extended functions and applications of such plasmonic nanodevices.

In our experiments, an optical fiber longer than 20 cm has been employed, where the optical switching signals have to experience big dispersion due to the long‐distance propagation through the fiber after being produced at the AuNW‐grating‐integrated end surface. It needs to be noted further that optical nonlinearity of the fiber material may also introduce POS signals, since part of the pump pulses were coupled into the fiber together with the probe. However, our experimental results on pure optical fibers without AuNW‐grating structures show that the corresponding transient absorption signals were always small and broad‐banded, which did not exhibit features disturbing those of the plasmonic response of the AuNWs. Nevertheless, the dispersion of the optical response of the long optical fibers may reduce the contrast and amplitude of the optical switching signals in the visible spectrum. Above effects may be observed in the transient spectroscopic performance of the optical switching signals.

### 2.2 Fabrication of the AuNW Grating on the Tip of an Optical Fiber

**Figure 2:**
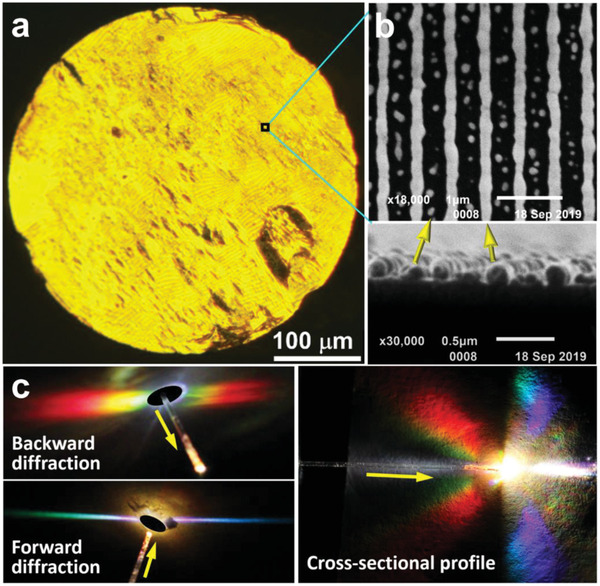
a) The optical microscopic image of the end surface of the optical fiber after being transferred with the grating structures and annealed at 450 °C. b) Top‐surface (upper panel) and cross‐sectional (lower panel) view SEM images of the AuNW grating on the fiber end. c) Diffraction patterns by the AuNW grating structures when a white‐light beam was coupled into the fiber at the unstructured end.

Interference lithography was first employed to produce a template photoresist (PR) grating with a period of about 520 nm and a modulation depth as large as 430 nm, as shown in Figure S1 in the Supporting Information. Large modulation depth of the PR grating enables confinement of large amount of colloidal gold nanoparticles during the annealing process, producing thick gold nanowires.^[^ ^38^ ^]^ After being annealed at 450 °C for 30 min, a gold nanowire grating was produced on the indium‐tin‐oxide (ITO) coated glass substrate. Then, the AuNW grating was transferred onto the end surface of an optical fiber by the flexible technique,^[^ ^37^ ^]^ as depicted in Figure S2 in the Supporting Information. The transferred fiber tip was then annealed at 450 °C, so that the AuNWs are fixed strongly on the fiber end surface, like being soldered onto the fiber tip. Meanwhile, the polymethyl methacrylate (PMMA) buffer layer employed during the flexible transfer process was removed by such an annealing process. **Figure** **2a** shows the optical microscopic image of the structured fiber end surface. The yellow color observed under the optical microscope is due to the diffraction by the grating. Figure 2b shows the scanning electron microscopy (SEM) image of a local area by the top‐surface (upper panel) and cross‐sectional (lower panel) views of the AuNWs on the planar substrate before they are transferred, showing continuous gold nanowires that have a thickness of 200 nm and a width of 190 nm in average. Excellent homogeneity of the grating structures and large thickness of the gold nanowires led to strong diffraction of light. Figure 2c shows the diffraction patterns at the structured fiber end, when a white light beam was coupled into the fiber from the unstructured end, where the backward (at longer wavelengths), forward (at shorter wavelengths), and cross‐sectional diffraction patterns are presented on the left‐top, left‐bottom, and right panels, respectively. The symmetry and high brightness of the diffraction patterns imply high quality of the transferred AuNW grating.

## 3 Ultrafast Optical Switching Performance

### 3.1 Plasmon Resonance of the AuNWs on Fiber End Facets

**Figure 3:**
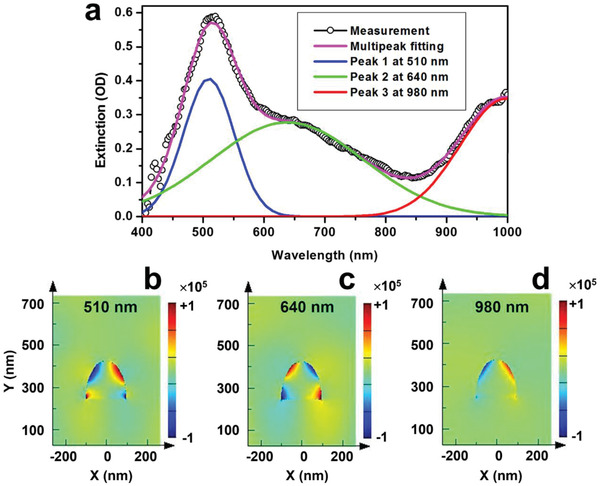
a) Optical extinction spectrum (open circles) measured on the gold nanowire grating integrated onto the end tip of an optical fiber and its comparison with the multipeak fitting using Gaussian functions. b–d) Calculated charge carrier density distribution in a gold nanowire (cross‐sectional profile) excited at 510, 640, and 980 nm, respectively, showing quadru‐ and dipolar plasmons.

**Figure** **3a** shows the measured optical extinction spectrum for transverse‐magnetic (TM) polarization by the open circles. In the measurement, a white light beam was coupled into the optical fiber through the structured end with the AuNW grating and the transmission output spectrum *I*(*λ*) was measured on the other unstructured end, which carries the optical response of plasmonic resonance of the AuNWs. A blank spectrum *I*_0_(*λ*) was measured by replacing the end‐structured optical fiber with an unstructured one. Thus, the optical extinction spectrum was calculated by −log_10_[*I*(*λ*)/*I*_0_(*λ*)]. Due to the limited range of the measurable spectrum of the spectrometer, the measured spectrum is plotted between 400 and 1000 nm in Figure 3a.

Decomposing the optical extinction spectrum in Figure 3a into multiple Gaussian functions, we may resolve three spectral features, which are peaked at about 510, 640, and 980 nm, as shown by the blue, green, and red spectra in Figure 3a, respectively. The overlap of these three spectra fits very well the measured spectrum, as shown by the magenta curve, implying precise resolving of the involved resonance modes. For understanding the mechanisms, we calculated the charge distribution for excitations at 510, 640, and 980 nm in a gold nanowire in the 2D profiles, as shown in Figure 3b–d, respectively, where we have assumed semielliptical shapes. Clearly, the spectral feature around 980 nm results from a dipolar plasmon and those at about 510 and 640 nm correspond to two different quadrupolar plasmons, which are based on the large thickness of the gold nanowires. However, the resonance mode at 510 nm is mainly located on the top and that in the red is closer to the bottom of the gold nanowire. Thus, the localized surface plasmon at 510 nm is more like a dipolar resonance on the upper surface with weak induction of oscillating charge carriers on the lower edges of gold nanowires. In contrast, the one at 640 nm is a quadrupolar mode with surface charges over the whole gold nanowire taking part in the plasmonic process, as shown clearly in Figure 3c.

It needs to be noted that interband transition for gold takes place at about 500 nm and a spectral dip is generally observed in the optical extinction response. However, in Figure 3a, we observe a strong peak at about 510 nm in the optical extinction spectrum of the gold nanowires, agreeing well with the proposed mechanism of higher‐order localized surface plasmons. These plasmonic features lay basis for the optical switching performance of the structured fiber end.

According Figure 3a, the AuNWs bring about high insertion loss, which is about 0.5 OD at maximum and is a typical value for strong localized surface plasmons. Plasmonic scattering of light by AuNWs has been the main mechanism. However, optical switching signal is dependent on the shift of the band‐edge of the resonance spectrum, therefore, steeper band‐edge favors stronger optical switching signals. On such basis, strong optical extinction by surface plasmons with strong spectral intensity and narrow spectral bandwidths are expected to achieve strong optical switching signals. In our case, the bandwidth of the resonance spectrum at full width at half maximum (FWHM) is about 100 nm, according to Figure 3a, which is a good configuration that favors optical switching performance.

### 3.2 Ultrafast POS Signals Detected at the Output End of the Fiber

**Figure 4:**
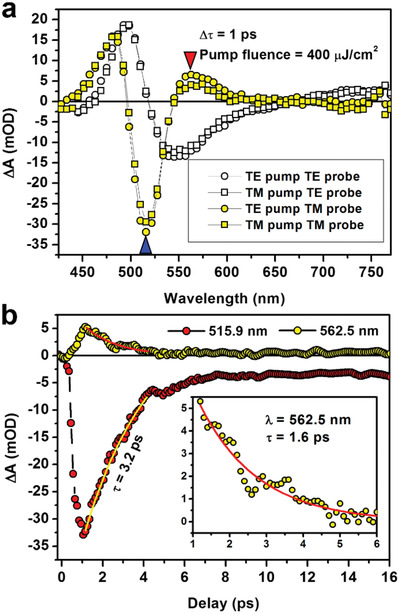
a) TA spectra at a delay of 1 ps for different polarization directions of the pump and probe pulses. b) TA dynamics for TE pump TM probe at 515.9 (red) and 562.5 nm (yellow) with fittings by first‐order exponential decay functions. Inset: Enlarged view of the dynamics curve at 562.5 nm.

As described in Section 2.1, the ultrafast optical switch delivered by an optical fiber was investigated using femtosecond TA spectroscopy, where the experimental geometry is shown schematically in Figure 1a. The structured end of the optical fiber was fixed such that the gold nanowires are oriented vertically, so that we can employ different polarization configurations of the pump and probe pulses. In all of the TA measurements, we have used a pump fluence of 400 µJ cm^−2^. **Figure** **4a** shows the TA spectra measured at the unstructured end of the optical fiber for four different polarization combinations of the pump and probe pulses at a delay of 1 ps, where the TA signals reach their peak amplitudes.

Clearly, the TA spectra in Figure 4a have almost the same amplitude and shape for the same polarization of the probe pulses, implying very little dependence on the polarization of the pump. For transverse‐electric (TE) polarization, which is along the extending direction of the gold nanowires, TA signal goes across the wavelength axis at 518 and 660 nm. Thus, a negative TA spectrum is observed in the range from 518 to 660 nm, positive spectra are observed at wavelength shorter than 518 nm and longer than 660 nm.

It is understandable that no localized surface plasmon was excited at TE polarization. However, strong band‐edge effects can be excited by femtosecond pump–probe detection, which have been investigated in detail in our previous work.^[^ ^39^ ^]^ Furthermore, the remaining gold nanoparticles between the gold nanowires and the inhomogeneity of the gold nanowires, as shown in the SEM image in Figure 2b, led to LSPR modes at TE polarization, which has weak modulation on the TA spectrum. Therefore, the TA spectra probed at TE polarization have been based mainly on the quenching of LSPR by strong optical excitation induced intraband transition, as depicted in Figure 1b–d.

For TM polarization, the TA spectra exhibit complicated features, which are completely different from those for TE. These differences resulted from strong localized surface plasmon resonance of the gold nanowires, or the quadrupolar plasmons in the visible spectrum. Interaction between localized surface plasmons in the gold nanowires and the band‐edge modulation in gold by femtosecond laser pulses induced complicated TA spectroscopic response. Three zero points can be observed with the TA spectra, which are located at 497, 545, and 650 nm. A much narrower strong negative spectrum is observed between 497 and 545 nm, as compared with the negative spectrum for TE polarization. This can be interpreted dominantly as the “quenching” of localized surface plasmon by the band‐edge shift effect under strong optical excitation, as explained in Figure 1b–d, where the optical extinction due to the original localized surface plasmon was much reduced or quenched, resulting in a strong negative TA spectrum. These mechanisms explain the zero points at 497 and 545 nm. Thus, interband absorption is enhanced for wavelength shorter than 497 nm, producing the positive spectrum peaked at 486.6 nm; plasmonic absorption/scattering is reduced between 497 and 550 nm, producing the strong negative spectrum dipped at 519.5 nm (blue triangle), which can be taken as a “quenching” effect of LSPR by band‐edge shift; redshifted plasmon enhances absorption/scattering at wavelength longer than 550 nm, producing a positive spectral feature peaked at 562.5 nm (red triangle), as shown in Figure 4b. Furthermore, strong optical excitation induced redshift of the localized surface plasmons peaked at about 640 and 980 nm led to weak and complicate variation of the TA spectrum in the range from 600 to longer wavelengths, producing the zero point at about 650 nm.

More specifically, since no LSPR is involved in the TE‐probed TA spectrum, nearly pure plasmonic quenching effect can be observed in Figure 4a by the black circles and squares, where a positive signal is observed on the left and a negative on the right in the TA spectrum. The corresponding left band‐edge is the falling edge of the interband absorption spectrum and the right is the rising edge of the plasmon band. In contrast, for TM‐probing, the redshift of LSPR in the AuNWs under strong optical excitation modulated the TA spectrum further, which resulted from enhanced electron–electron and electron–phonon scattering processes and led to the redshift of the whole resonance spectrum. As a result, the TA spectrum for TM polarization combined the “left‐positive right‐negative” signal on the shorter‐wavelength region for the quenching effect and the “left‐negative right‐positive” signal on the longer‐wavelength region for the redshifted LSPR, as can be resolved clearly in Figure 4a by the yellow‐filled squares and circles.

In Figure 4b, we plot the TA dynamics for TE pump and TM probe at 515.9 and 562.5 nm in a delay time range of 0–16 ps, which corresponds to the peak values of the negative and positive TA spectra, as highlighted by blue and red triangles, respectively, in Figure 4a. The amplitude of the negative spectrum is about 34 mOD at 515.9 nm, corresponding to an enhancement of the transmission by about 7.6% through the fiber, whereas, the amplitude becomes smaller than 7 mOD at 562.5 nm. These two dynamic curves are the optical switching signals delivered to the unstructured end of the fiber. Fitting the two dynamic curves in Figure 4b using first‐order exponential decay functions, we can resolve the lifetimes of the optical switching processes. Considering the much smaller amplitude at 562.5 nm, the TA dynamics is enlarged in the inset of Figure 4b. According to the fittings, the negative signal has a lifetime of about 3.2 ps, which agrees well with the lifetime of band‐edge plasmons, as has been evaluated in ref. ^[^ ^39^ ^]^. The positive signal has a lifetime of shorter than 1.6 ps and is based on the interaction of plasmonic electrons and phonons.

Efficiency of the ultrafast POS device is an important index to evaluate the optical modulation performance, in particular for the device on an optical fiber tip with respect to those on planar substrates. We may define the efficiency by the ratio of the spectroscopic modulation depth over the pump fluence as: *η* = Δ*A*_max_/*P*_f_, where Δ*A*_max_ is the amplitude of the TA spectrum and *P*_f_ is the pump fluence by mJ cm^−2^. For this fiber‐delivered POS device, we have *η*_fiber_ = 85 mOD cm^2^ mJ^−1^ at 515.9 nm. Using the same definition, we may calculate the efficiency of the excellent ultrafast POS device reported in refs. ^[^ ^23^, ^40^ ^]^, which are about 79 and 53 mOD cm^2^ mJ^−1^, respectively. Therefore, the fiber‐delivered device even shows higher efficiency in ultrafast optical modulation than those fabricated on planar substrates. In other words, low driving threshold is achieved with such a device.

It needs to be stressed further that one of the important advantages of the gold nanodevices is their high stability with high resistance to thermal and chemical damage mechanisms. In our studies on the ultrafast optical modulation performance, we did not observe any obvious morphology changes due to strong optical irradiation and the transient spectroscopic response stays stably over the whole measurement procedures. In Figure S3 in the Supporting Information, we present experimental data to verify the stability of the AuNW grating under continuous illumination of femtosecond laser pulses for nearly 3 h (correspond to the continuous excitation by more than 10^7^ laser pulses).

Moreover, the homogeneity of the AuNW grating on the fiber end facet is also important for the working performance of the POS device. Figure S4 in the Supporting Information includes a series of TA measurements to show reproduced ultrafast optical switching properties on different locations. Figure S4a in the Supporting Information shows how the different locations of the probe laser spots were selected. The measurement results are presented in Figure S4b,c in the Supporting Information by the TA spectra and TA dynamics, respectively, for TM and TE polarizations. Clearly, the transient spectroscopic response can be well reproduced on different locations, verifying the excellent homogeneity of the structures on the fiber end surface. In particular, for different locations, the orientation of the probe laser beam was also slightly adjusted. The excellent reproducibility of the TA signals also implies insensitivity of the POS device to the small change of the angle of incidence, which favors practical applications of such a system.

## 4 Conclusions

We report in this work an ultrafast optical switch integrated on the end facet of an optical fiber. The optical switching signal generated in the plasmonic nanostructures may be delivered by the fiber waveguide to the output end over a long propagation distance. Interaction between localized surface plasmon in the gold nanowires and the band‐edge plasmon induced by femtosecond laser pulse excitation led to a quenching effect of LSPR and is responsible for the optical switching effects. An on/off speed higher than 3.2 ps and a modulation depth of about 7.6% on the transmission through the optical fiber have been achieved for the optical switch. Such a plasmonic fiber device makes significant contribution to the lab‐on‐fiber techniques and implies potential applications in optical communication systems. Furthermore, miniaturization of the optical switching device onto the fiber tip with high success rate is important for integrated optics. High‐quality structures covering the whole planar surface of the fiber end implies full optical modulation of the input with high signal‐to‐noise ratios and advantages over the side‐surface design. Direct integration onto the end facets of fibers enables high coupling efficiency, automatic mode matching between the input and transmission, as well as easy incorporation with fiber lasers and other fiber‐based data processing channels for implementing all‐fiber systems.

## Conflict of Interest

The authors declare no conflict of interest.

## Supporting information

Supporting Information

## Data Availability

Research data are not shared.
