# Supplementary material for: Optical Fiber Delivered Ultrafast Plasmonic Optical Switch
Source: Adv Sci (Weinh). 2021 Mar 16;8(10):2100280. doi: 10.1002/advs.202100280 (PMC8132049; doi:10.1002/advs.202100280)
Supplement: Supplementary file 1 — Supporting Information [file ADVS-8-2100280-s001.pdf]

© 2021 Wiley-VCH GmbH

ADVANCED SCIENCE
Open Access

Supporting Information

for *Adv. Sci.*, DOI: 10.1002/advs.202100280

Optical Fiber Delivered Ultrafast Plasmonic Optical Switch

Jinghui Yang[1,2] *and Xinping Zhang*[1,*]

Supporting Information

Optical fiber delivered ultrafast plasmonic optical switch

Jinghui Yang[1,2] and Xinping Zhang[1,*]

[1] *Institute of Information Photonics Technology, Faculty of Science, Beijing University of Technology, Beijing 100124, China*

[2] *Modern Police Technology and Equipment Research Center, College of Police Equipment and Technology, China People's Police University, Langfang 065000, China*

*Email: zhangxinping@bjut.edu.cn

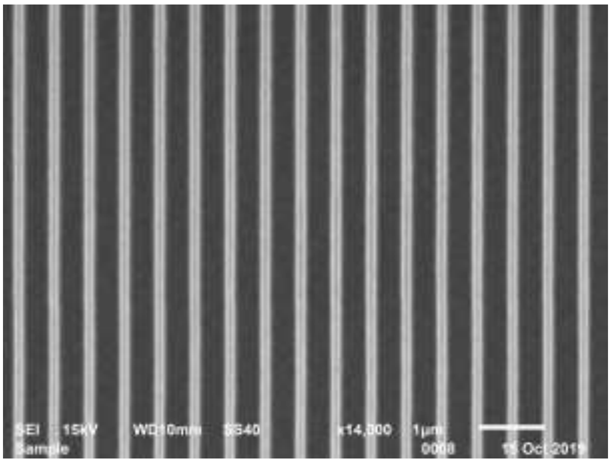

(a)

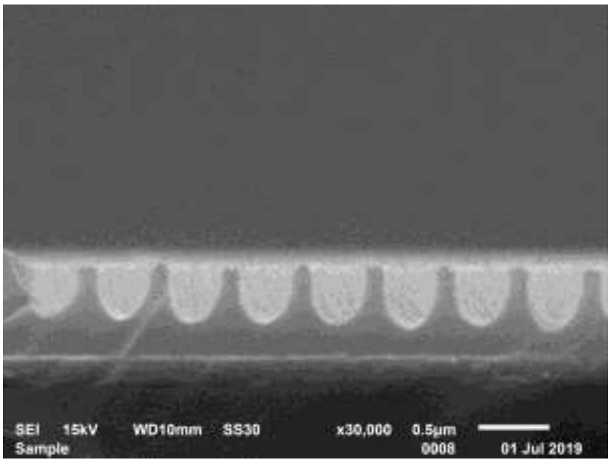

(b)

Fig. S1 Microscopic characterization of the template photoresist (PR) grating. (a) SEM image of the photoresist grating structures fabricated on an ITO-coated glass substrate with a period of about 520 nm. (b) The SEM of the cross-sectional profile of the PR grating.

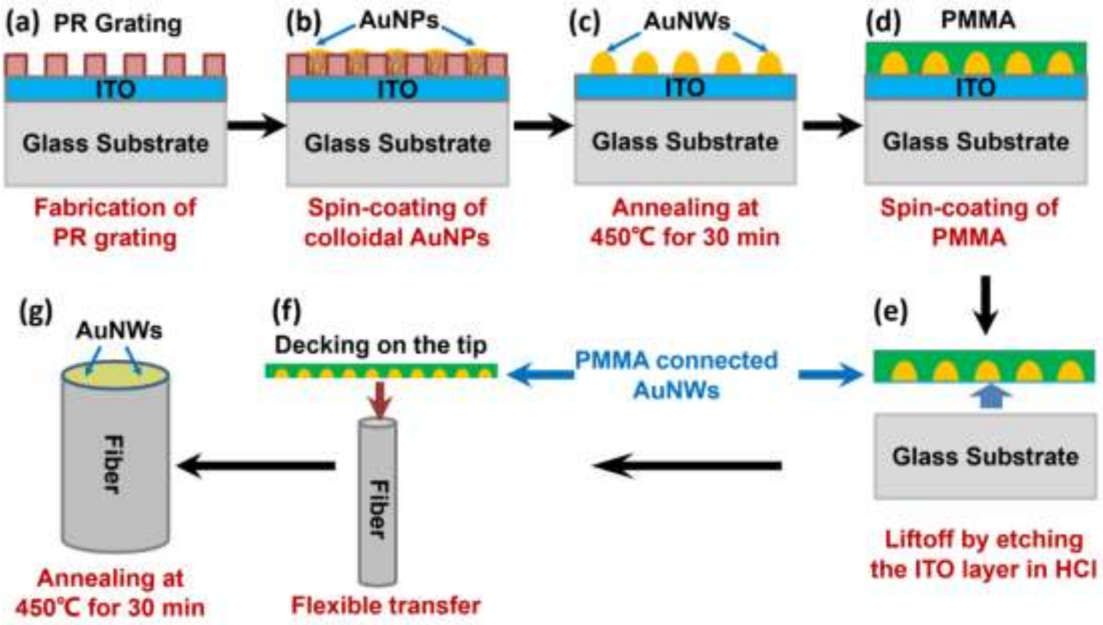

Fig. S2 Fabrication procedures for the gold nanowire (AuNW) grating on the fiber tip. (a) Preparation of a photoresist (PR) grating on an ITO-coated glass substrate using interference lithography. (b) Spin-coating of colloidal gold nanoparticles (AuNPs). (c) Annealing at 450 °C for 30 min to produce gold nanowires and to remove the PR. (d) Spin-coating of PMMA to produce a buffer layer on the gold nanowires. (e) Liftoff of PMMA connected AuNWs from the glass substrate by etching the ITO layer in HCl. (f) Flexible transfer of the AuNWs onto the end facet of the optical fiber. (g) Annealing at 450 °C for 30 min to fix the AuNWs and to remove the PMMA.

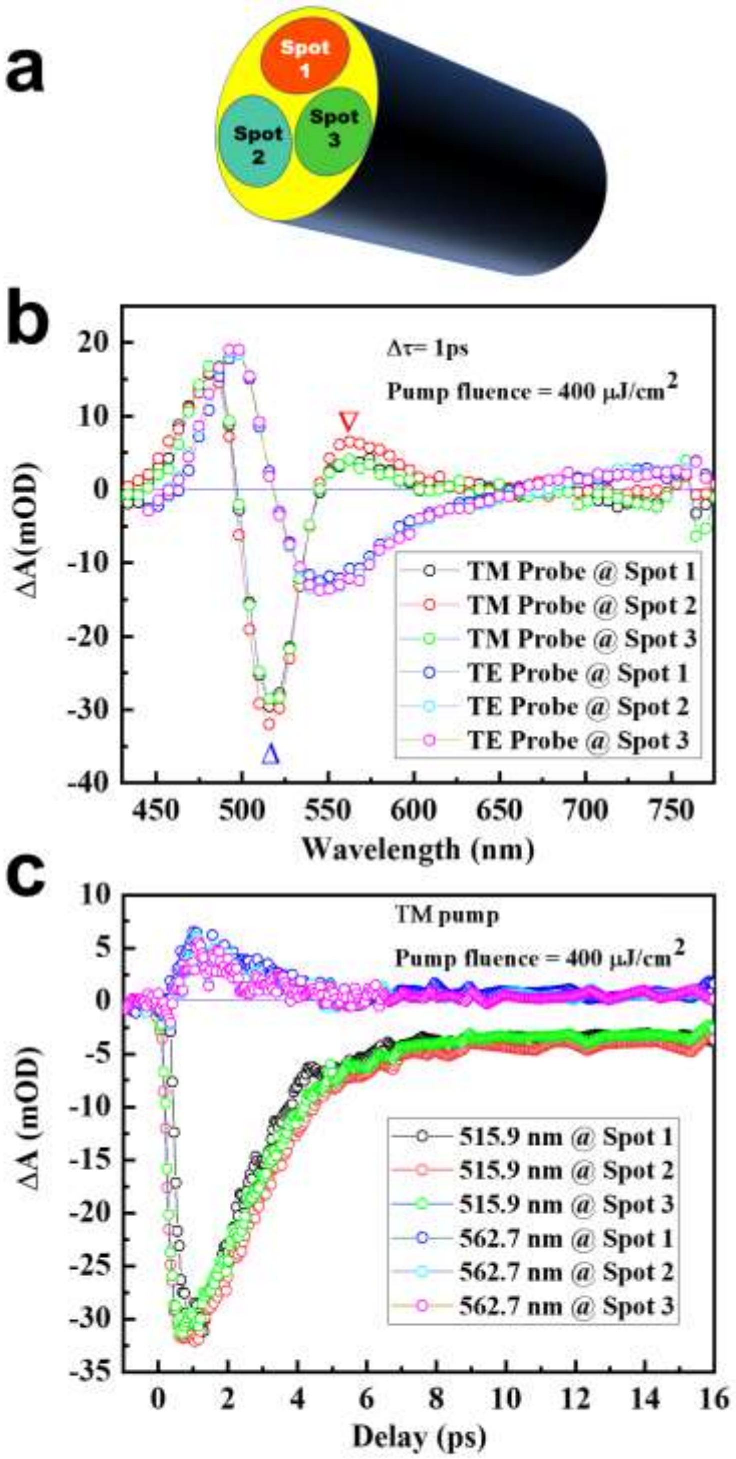

Fig. S3 Verification of the spatial homogeneity of the ultrafast optical switching device over the surface of the end facet of an optical fiber. (a) Schematic illustration of three different positions of the probe laser spots on the fiber end facets for different measurements. (b) TA spectra measured for TE and TM polarizations at different positions on the fiber end facet, as specified in (a). (c) TA dynamics measured 515.9 and 562.7 nm at different positions on the fiber end facet, as specified in (a).

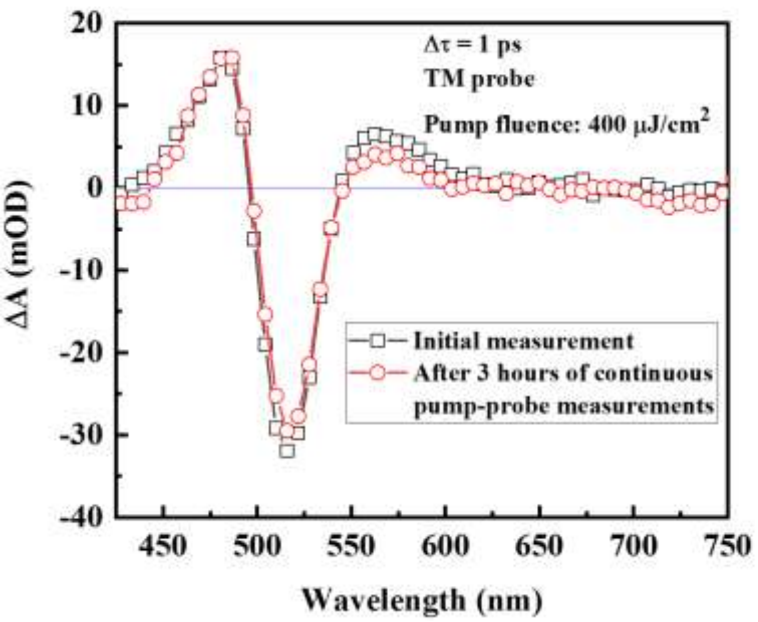

Fig. S4 Long-term stability of the fiber-delivered ultrafast optical switch: TA spectra measured on the fresh sample (black squares) and on the same sample after 3 hours of continuous pump-probe (red circles), corresponding to about 8-9 full sweeps of the delay stage of the TA measurement system.
